# A Meta-Analysis to Assess the Probable Association of Hypertension During Pregnancy and Placenta Accreta

**DOI:** 10.3390/medicina61020297

**Published:** 2025-02-08

**Authors:** Mohamed S. Imam, Dina Meshari Abdularhman Alnaim, Renad Khalid Abdullah Alaraifi, Juman Salah Saleh Alabduljabbar, Alanoud Abdulrahman Mohammed Alhamed, Asalah Mohammed Fayadh Alansari, Raghad Abdullah Ali Alqarni, Shouq Fahad Saleh Alotaibi, Dimah Zuwayyid Aali Alsufyani, Rana Mohammed Abdullah Alzaidi, Shahad Ali Hussain Mathkoor, Rawabi Hameed Hamde Alotaibi, Mohamed E. A. Abdelrahim, Basma M. E. Mohamed

**Affiliations:** 1 Department of Clinical Pharmacy, National Cancer Institute, Cairo University, Fom El Khalig Square, Kasr Al-Aini Street, Cairo 11796, Egypt; soliman@su.edu.sa; 2 College of Clinical Pharmacy, King Faisal University, Al Hofuf 31982, Al-Ahsa, Saudi Arabia; 222437935@student.kfu.edu.sa (D.M.A.A.); 222431914@student.kfu.edu.sa (R.K.A.A.); 3 College of Pharmacy, Qassim University, Buraidah 51452, Qassim, Saudi Arabia; 351200389@qu.edu.sa (J.S.S.A.); 342216014@qu.edu.sa (A.A.M.A.); 4 College of Pharmacy, Taif University, Taif 21944, Mecca, Saudi Arabia; s44110095@students.tu.edu.sa (A.M.F.A.); s44110079@students.tu.edu.sa (R.A.A.A.); s44110089@students.tu.edu.sa (S.F.S.A.); s44110099@students.tu.edu.sa (D.Z.A.A.); s44103080@students.tu.edu.sa (R.M.A.A.); s44110003@students.tu.edu.sa (S.A.H.M.); s44110065@students.tu.edu.sa (R.H.H.A.); 5 Clinical Pharmacy Department, Faculty of Pharmacy, Beni-Suef University, Beni-Suef 62574, Egypt; mohamed.abdelrahim@pharm.bsu.edu.eg

**Keywords:** hypertension, pregnancy, placenta accreta

## Abstract

*Background and Objectives*: A meta-analysis was conducted to assess the probable association of hypertension during pregnancy and placenta accreta (PA). *Materials and Methods*: A systematic literature search was conducted up to November 2024, resulting in the identification of 10 studies encompassing 128,589 pregnant women. They reported associations between the possible impacts of hypertension during pregnancy and PA. The odds ratio (OR), with 95% confidence intervals (CIs), was computed to evaluate the possible association of hypertension during pregnancy and PA, utilizing a dichotomous approach with either a random or fixed-effect model. *Results*: No significant difference was found between hypertension during pregnancy and control (no hypertension during pregnancy) in the occurrence of PA (OR, 0.74; 95% CI, 0.52–1.04, *p* = 0.08). Also, no significant difference was found between pregnant women with PA and control (no PA) in the occurrence of hypertension (OR, 1.15; 95% CI, 0.61–2.19, *p* = 0.66). *Conclusions*: Hypertension during pregnancy has no impact on the occurrence of PA, and vice versa. More research is desired to approve these outcomes.

## 1. Background

One of the serious pregnancy problems that can result in severe and probably fatal hemorrhages during and after delivery is placenta accreta (PA) [1,2]. With a frequency of 1:500 births for the years 1982–2002 in the USA, PA has become significantly more common in recent years, suggesting the rising rate of emergency hysterectomy and cesarean delivery [3]. There is some evidence linking PA to some risk factors, e.g., advanced maternal age, placenta previa, and previous cesarean section [4,5]. Previous research indicated that PA and pregnant hypertension were positively correlated [6]. However, another study has revealed contradictory consequences [7]. According to Bowman et al., women without PA were trending toward higher levels of hypertension [8]. Other studies indicated a decrease, although no significant trend was observed regarding PA in relation to hypertension during pregnancy [7,9]. The current meta-analysis study was to assess the probable association between hypertension during pregnancy and PA.

## 2. Methods

### 2.1. Eligibility Criteria

The present meta-analysis complies with the epidemiology statement’s meta-analysis of papers (PRISMA) [10]. This was carried out by a set protocol.

### 2.2. Information Sources

**Figure 1:**
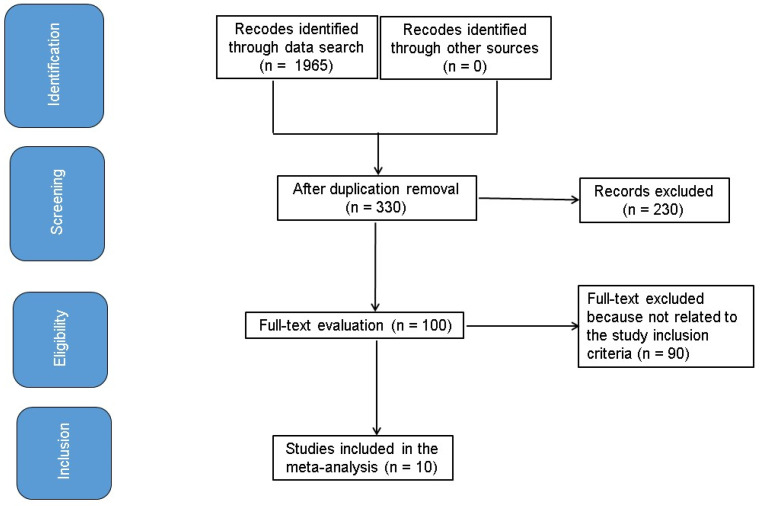
Schematic diagram of the study procedure.

Figure 1 illustrates the study methodology.

The criteria for including studies were as follows:

1. The research was retrospective study.
2. The focus of the populace was pregnant women.
3. The study examined hypertension during pregnancy.
4. The study provided the probable association between hypertension during pregnancy and PA.

The criteria for excluding studies were as follows:

1. Research that did not involve a comparison between hypertensive pregnant women and a control group.
2. Studies concerning conditions unrelated to PA.
3. Research that did not emphasize the impact of comparative findings.

### 2.3. Search Strategy

A set of search strategies was developed based on the PICOS framework [11]. The population (P) consists of pregnant women; the intervention/exposure (I) is hypertension during pregnancy; the comparison (C) includes hypertension during pregnancy and a control group of no hypertension during pregnancy; the outcome (O) refers to PA [12].

Initially, a systematic search was performed across OVID, Embase, Google Scholar, Cochrane Library, and PubMed up to December 2024, utilizing a combination of keywords as in Table 1. All studies recognized were composed in an EndNote file, duplicates were eliminated, and the titles and abstracts were assessed to exclude studies that did not demonstrate any one probable connotation of hypertension during pregnancy and PA according to pre-established inclusion and exclusion criteria. The lasting studies were then reviewed for relevant information.

### 2.4. Selection Process

The primary objective was to measure the probable association of hypertension during pregnancy and PA. A summary was compiled based on the appraisal of the possible probable association of hypertension during pregnancy and PA.

### 2.5. Data Collection Process

The study assessed the probable association of hypertension during pregnancy and PA. Only human research published in English was deemed eligible for inclusion. There were no restrictions based on the type or size of the study. Studies excluded from consideration included review articles, commentaries, and those that did not present a level of connotation.

### 2.6. Data Items

In this research, the main consequence of the inclusion parameter was analyzed. There were no restrictions on the quantity of subjects that could be found for the research. Since letters, reviews, and editorials do not present a position in the meta-analysis, we did not integrate these kinds into our creation.

### 2.7. Study Risk of Bias Assessment

Due to the probable for bias in the studies, two writers independently evaluated the methodological quality of the chosen investigations. The RoB 2 tool, an updated Cochrane risk-of-bias instrument for randomized trials, was employed to assess methodological quality [13]. Regarding the valuation criteria, each study was evaluated and categorized into one of three levels of bias risk: low, if all quality criteria were fulfilled; unclear, if one or more quality criteria were partially met or ambiguous, thus representing a moderate risk of bias; or high, if one or more criteria were not fulfilled or absent, marking the study as having a high risk of bias. Any discrepancies were resolved by reexamining the original article.

### 2.8. Effect Estimates

Sensitivity analyses were confined to research illustrating the association between the probable association of hypertension during pregnancy and PA. In the subgroup and sensitivity analysis, hypertension during pregnancy was compared to a control group of normotensive pregnant women.

### 2.9. Synthesis Methods

The odds ratio (OR) along with the 95% confidence interval (CI) was determined using the dichotomous method with either a random or fixed-effect model. The I2 index was computed, with values ranging from 0% to 100%. An I2 index of approximately 0%, 25%, 50%, and 75% indicates no, low, moderate, and high levels of heterogeneity, respectively [11]. If the I2 value exceeded 50%, we employed the random-effects model; if it fell below 50%, we opted for the fixed-effects model. The initial evaluation was categorized based on the outcomes as previously outlined to perform the subgroup analysis. A *p*-value below 0.05 was employed to ascertain statistically significant differences among the subcategories.

### 2.10. Reporting Bias Assessment

The presence of studies bias was assessed quantitatively using the Egger regression test (with studies bias indicated if *p* ≥ 0.05), and qualitatively through a visual inspection of the funnel plots, which displayed the logarithm of the odds ratios against their standard errors [14].

### 2.11. Certainty Assessment

All *p*-values were determined using two-tailed tests. Calculations and graphs were completed using Reviewer Manager version 5.3 (The Nordic Cochrane Centre, The Cochrane Collaboration, Copenhagen, Denmark).

## 3. Results

In total, 10 studies were chosen for the meta-analysis out of a possible 1965 (between 1999 and 2014) met the requirements for inclusion and were added to the study [6,7,8,9,15,16,17,18,19,20].

**Figure 2:**
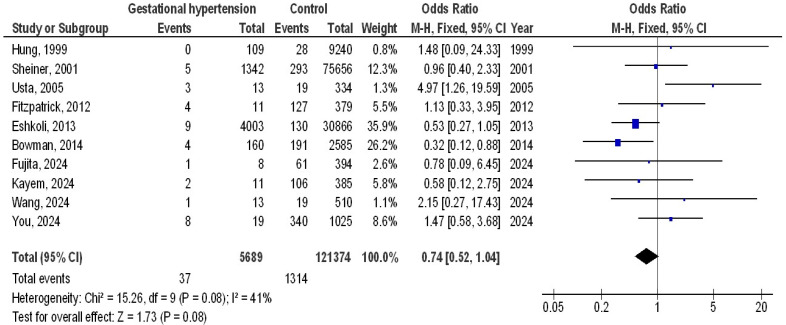
Forest plot of the hypertension during pregnancy compared to no hypertension in pregnancy with the occurrence of placenta accreta [6,7,8,9,15,16,17,18,19,20].

**Figure 3:**
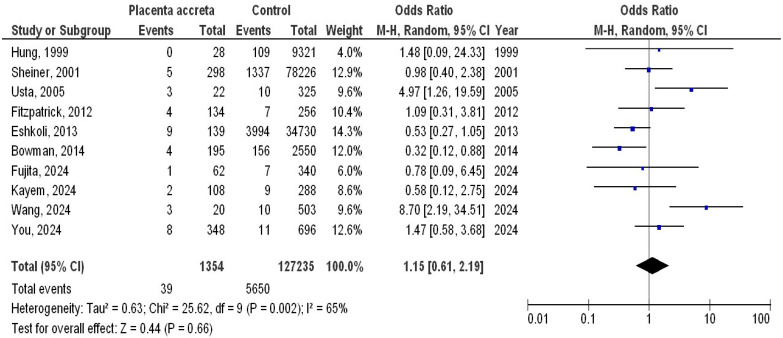
Forest plot of the pregnant women with placenta accreta compared to no placenta accreta with the occurrence of hypertension in pregnancy [6,7,8,9,15,16,17,18,19,20].

A total of 128,589 pregnant women were enrolled in the 10 studies; 4225 of them had hypertension throughout pregnancy, and 1354 of them had PA. The 10 studies discussed the possible connections between PA and hypertension during pregnancy. The number of pregnant women in the selected studies varied from 334 to 78,524. Table 2 displays the specifics of the 10 studies. As illustrated in Figure 2, the occurrence of PA (OR, 0.74; 95% CI, 0.52–1.04, *p* = 0.08) with low heterogeneity (I2 = 41%) did not differ significantly between hypertension during pregnancy and control (no hypertension during pregnancy). Additionally, as illustrated in Figure 3, there was no significant difference in the occurrence of hypertension between pregnant women with PA and control (no PA) (OR, 1.15; 95% CI, 0.61–2.19, *p* = 0.66), with moderate heterogeneity (I2 = 65%).

**Figure 4:**
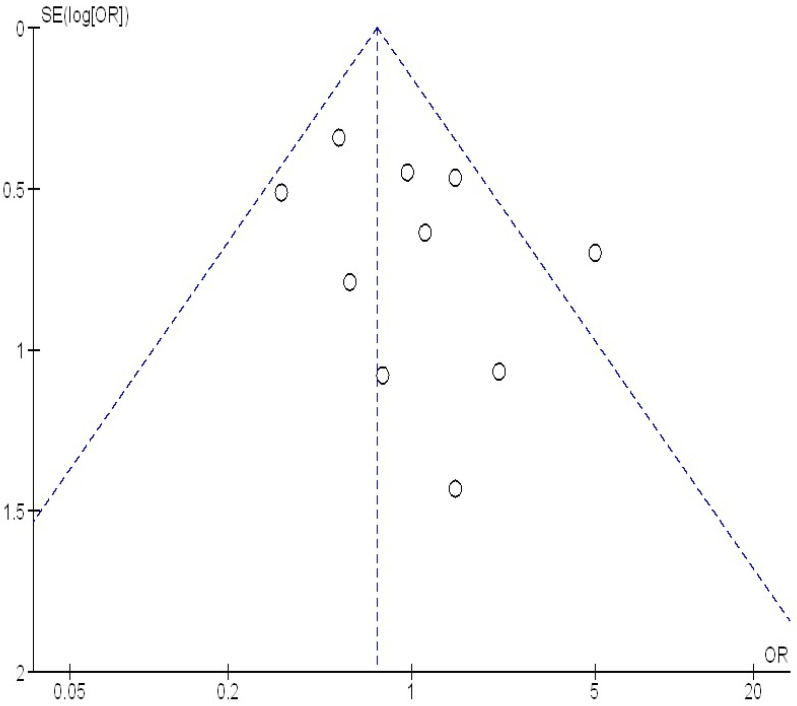
Funnel plot of the hypertension during pregnancy compared to no hypertension in pregnancy with the occurrence of placenta accreta.

**Figure 5:**
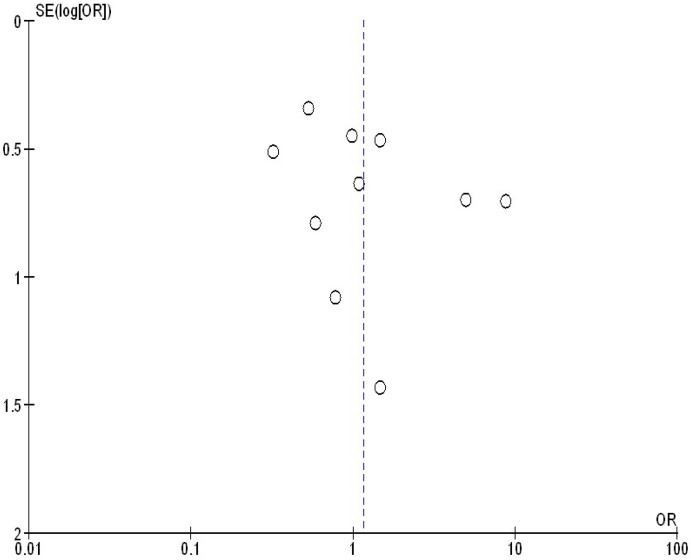
Funnel plot of the pregnant women with placenta accreta compared to no placenta accreta with the occurrence of hypertension in pregnancy.

As shown in Figure 4 and Figure 5, there was no indication of studies bias based on both the visual inspection of the funnel plot and the quantitative measurement utilizing the Egger regression test (*p* = 0.89). Nevertheless, because of their small sample size, the majority of the encompassed studies were believed to have poor methodological quality. Selective reporting bias was absent from every study, and no papers had selective reporting or insufficient outcome data.

## 4. Discussion

This study depended on 10 studies encompassing 128,589 pregnant women [6,7,8,9,15,16,17,18,19,20]. The occurrence of PA did not significantly differ between hypertension during pregnancy and control (no hypertension during pregnancy). Additionally, there was no significant difference in the occurrence of hypertension between pregnant women with PA and control (no PA) [7,8,9,15,16,17,18,19,20,21]. Preeclampsia, eclampsia, and chronic and gestational hypertension are among the conditions that fall under the category of hypertension during pregnancy. The last three conditions are temporary hypertensive conditions that start during pregnancy [22]. Hypertension during pregnancy pathogenesis is not fully understood [23]. The exact pathophysiology of hypertension during pregnancy remains unknown. Reduced interaction between endometrium/decidua and trophoblasts might be a probable cause [24]. Since the majority of women who come with PA had prior curettage or cesarean births, placenta tissue damage from previous equipment is thought to have a part in the condition [24]. The costs of earlier instrumentation are thought to include unneeded trophoblastic invasion, abnormal maternal vascular remodeling, or imperfect decidualization [17]. According to a prior study, PA was more common in pregnant mothers with hypertension [6]. However, two additional larger studies found no significant correlation between PA and hypertension during pregnancy, and both found that women without PA had higher levels of hypertension [7,9]. Women without PA also had higher levels of hypertension, according to Bowman et al. [8]. According to this meta-analysis, the risk of PA does not significantly decrease for hypertension during pregnancy. In hypertension during pregnancy, aberrant trophoblastic invasion may not reduce PA. In addition to affecting placenta implantation, the endothelial damage that leads to the hypertensive disorders of pregnancy. Hypertension during pregnancy and PA may be significantly influenced by certain regulatory T-cells. According to a study, preeclampsia is linked to a reduction in the quantity of regulatory T-cells [25]. Increased regulatory T-cell counts may be responsible for extravillous trophoblast over-invasion. Using immunohistochemistry, Schwede et al. showed that increased extravillous trophoblast invasion in PA may be linked to higher regulatory T-cells numbers [26]. Another indication of the detrimental connection between hypertension throughout pregnancy and PA is pregnancy-connected plasma protein-A. To predict the early onset of preeclampsia, Anna Yiniemi et al. assessed usefulness of a mixture of soluble tumor necrosis factor receptor-1, maternal characteristics, free human chorionic gonadotropin β, pregnancy-connected plasma protein-A, and alpha-fetoprotein. They found that lower levels of pregnancy-connected plasma protein-A were linked to an increased risk of developing preeclampsia [27]. Nonetheless, a positive correlation between PA and plasma protein-A linked to first-trimester pregnancy was previously demonstrated [28]. The contradictory results may indicate a link between PA and hypertension during pregnancy. Pregnancy requires modification of the uterine spiral arteries, placentation, trophoblast invasion of the maternal decidua, myometrium, and synchronized implantation of embryo into receptive decidua. Preeclampsia and PA are just two of the pregnancy issues that might result from skipping any of these steps [29]. Trophoblast invasion depends on the activity of the antiangiogenic molecule soluble fms-like tyrosine kinase. A secreted splice form of fms-like tyrosine kinase, soluble fms-like tyrosine kinase binds irreversibly to placental growth factor and circulating vascular endothelial growth factor. It is supposed to have an impact on preeclampsia pathogenesis [30]. However, soluble fms-like tyrosine kinase at the maternal-fetal boundary appears less frequently in women with PA than in those with normal placentation, which is a feature of their invasive trophoblasts [31]. PA and pregnant hypertension did not correlate, according to this meta-analysis. A previously published meta-analysis produced an opposing finding and suggested more research with a larger sample size [32]. They discovered a significant correlation between a decrease in PA and hypertension during pregnancy. The occurrence of hypertension during pregnancy in women with PA as opposed to control was not examined. A possible explanation for our results’ higher *p*-values than Wang et al.’s could be that we included ten studies instead of the three in their analysis. Nevertheless, additional studies are prerequisites to endorse these possible correlations and to produce a clinically significant change in the outcomes [33,34,35,36]. Larger, more uniform sample sizes and well-conducted studies are required for these investigations to analyze some additional factors, e.g., diverse women’s ages and ethnicities. Additionally, it was challenging to investigate the various forms of hypertension during pregnancy, such as preeclampsia superimposed upon chronic hypertension, and eclampsia, preeclampsia, and chronic hypertension complicating pregnancy, due to the small sample size and limited number of encompassed studies in the current meta-analysis.

## 5. Limitations

This meta-analysis may exhibit selection bias due to the exclusion of numerous identified studies. The excluded studies did not meet the meta-analysis inclusion criteria. Moreover, we were unable to ascertain if the outcomes were connected with women’s ethnicity and age. The study aimed to assess the probable connotation of hypertension during pregnancy and PA, relying on data from prior studies that may present bias due to inadequate information. Factors such as age, nutritional status, and ethnicity of patients were probable sources of bias. Unpublished studies and absent data might introduce bias in aggregated effects. Investigation of different types of hypertension during pregnancy, including eclampsia, chronic hypertension complicating pregnancy, preeclampsia superimposed on chronic hypertension, and preeclampsia, was hindered in the current meta-analysis by the limited number of studies and small sample sizes. A comprehensive examination of the impacts of PA, placenta increta, and placenta percreta was not feasible due to the absence of PA data in the selected articles.

## 6. Conclusions

Hypertension during pregnancy does not affect the occurrence of PA and PA in pregnancy does not affect the occurrence of hypertension. However, the evaluation of outcomes must be conducted with prudence due to the limited number of studies in our meta-analysis, indicating the need for extra research to corroborate these findings.

## Figures and Tables

**Table 1:** Search plan for each database.

Database	Search Strategy
Pubmed	#1 “placenta accreta” [MeSH Terms] OR “preeclampsia” [All Fields] OR “hypertension” [All Fields] #2 “pregnancy” [MeSH Terms] OR “placenta accreta” [All Fields] OR “morbidly adherent placenta” [All Fields] OR “gestation” [All Fields] #3 #1 AND #2
Embase	‘placenta accreta’/exp OR ‘preeclampsia’/exp OR ‘hypertension’/exp #2 ‘pregnancy’/exp OR ‘ICBG’/exp OR ‘morbidly adherent placenta’ OR ‘gestation’ #3 #1 AND #2
Cochrane Library	#1 (placenta accreta): ti,ab,kw OR (preeclampsia): ti,ab,kw OR (hypertension):ti, ab,kw (word variations were searched) #2 (pregnancy): ti,ab,kw OR (morbidly adherent placenta):ti,ab,kw OR (gestation):ti,ab,kw (word variations were searched) #3 #1 AND #2

**Table 2:** Attributes of the chosen studies for the meta-analysis.

Study	Country	Total	Placenta Accreta	Hypertension
Hung, 1999 [15]	Taiwan	9349	28	109
Sheiner, 2001 [16]	Palestine	78,524	298	1342
Usta, 2005 [6]	Lebanon	347	22	13
Fitzpatrick, 2012 [7]	UK	390	134	11
Eshkoli, 2013 [9]	Palestine	34,869	139	4003
Bowman, 2014 [8]	USA	2745	195	160
Kayem, 2024 [17]	France	396	108	11
You, 2024 [18]	China	1044	348	19
Wang, 2024 [19]	China	523	20	13
Fujita, 2024 [20]	Japan	402	62	8
	Total	128,589	1354	4225
